# Long-term prognosis and overall mortality in patients with progressive multifocal leukoencephalopathy

**DOI:** 10.1038/s41598-023-41147-9

**Published:** 2023-08-31

**Authors:** Jinnam Kim, Changhyup Kim, Jung Ah Lee, Se Ju Lee, Ki Hyun Lee, Jung Ho Kim, Jin Young Ahn, Su Jin Jeong, Nam Su Ku, Jun Yong Choi, Joon-Sup Yeom, Young Goo Song

**Affiliations:** 1 Division of Infectious Diseases, Department of Internal Medicine, Severance Hospital, Yonsei University College of Medicine, 50-1 Yonsei-Ro, Seodaemun-Gu, Seoul, 03722 Korea; 2 AIDS Research Institute, Yonsei University College of Medicine, Seoul, Korea; 3 Department of Internal Medicine, Hanyang University College of Medicine, Seoul, Korea

**Keywords:** Diseases of the nervous system, Infectious diseases, Viral infection

## Abstract

Progressive multifocal leukoencephalopathy (PML) is a rare but fatal opportunistic infection and mainly occurs in patients with immunosuppressive conditions. Despite the increasing number of patients receiving immunosuppressive treatments, studies on PML are still lacking due to its low prevalence and incidence. We retrospectively reviewed patients diagnosed with PML in two tertiary hospitals in South Korea from 1999 to 2021. Total of 47 PML patients were included. Of 27 patients (57.4%) were diagnosed with human immunodeficiency virus (HIV). Median last follow-up modified Rankin Scale (mRS) score was higher in the non-HIV PML group than that in the HIV group (5 vs. 4, *p* = 0.020). Median survival duration was lower in the non-HIV group (184 vs. 1,564 days). The 1-year and overall mortality rates of PML patients were significantly higher in the non-HIV group than that in HIV group (60.0% vs. 25.9%, *p* = 0.019; 80.0% vs. 40.7%, *p* = 0.007). Initial mRS score (HR 1.685, *p* = 0.038) and highly active antiretroviral therapy (HAART) in HIV patients (HR 0.374, *p* = 0.013) had a significant effect on overall mortality. Our findings suggest that early detection of PML with low mRS score and early initiation of HAART in patients with HIV may improve prognosis.

## Introduction

Progressive multifocal leukoencephalopathy (PML) is a demyelinating disease of the central nervous system (CNS) caused by reactivation of the John Cunningham virus (JCV), leading to oligodendrocyte destruction and subsequent demyelination^1^. PML is a rare but fatal opportunistic infection and mainly occurs in patients with immunosuppressive conditions, such as human immunodeficiency virus (HIV) infection, lymphoproliferative disorders, post-organ transplantation immunosuppression, and autoimmune diseases^1,2^. Several studies in the United States and Europe reported that 44–49% of patients were diagnosed with HIV, 22–31% with hematologic malignancies, 9–20% with chronic inflammatory diseases, and 3–4% with solid organ transplants^1,3,4^. Recently, PML has been associated with immunomodulatory medications such as natalizumab, efalizumab, and rituximab^5–7^.

Despite advances in treatment options such as pembrolizumab or BK virus-specific T cell-based immunotherapy, PML is a severe and fatal infection with a reported 1-year mortality rate of 38.2%^3,4,8^. Introduction of highly active antiretroviral therapy (HAART) increased the median survival of HIV patients from 0.4 to 1.8 years; however, the overall mortality rate remained at 74.5–78.0%^1,9^. Interestingly, in the non-HIV population, the median time to death after PML diagnosis was two months, and the case-fatality rate was 90%^10^.

Several potential therapeutic options have been used to improve this poor prognosis, but exhibited only a few clear benefits. Initiation of HAART in HIV patients has shown a clear survival benefit^11,12^ However, therapeutic agents like cytarabine, cidofovir, mefloquine, alpha-interferon, and serotonin antagonists have not shown potential in the prognosis of PML patients^13–17^. Therefore, it is necessary to study whether the recent development of HAART and changes in HAART initiation, regardless of CD4 cell count, will improve PML prognosis in HIV patients^18^. In addition, an increase in the number of patients receiving immunosuppressive treatments has led to PML occurrence in various immunosuppressive conditions^1,2^. Despite this, studies on PML are still lacking due to its low prevalence and incidence.

Therefore, this study aimed to investigate the long-term prognosis and prognostic factors for overall mortality of patients with PML through an extended observation period of over 21 years.

## Results

### Characteristics of patients with PML

**Figure 1:**
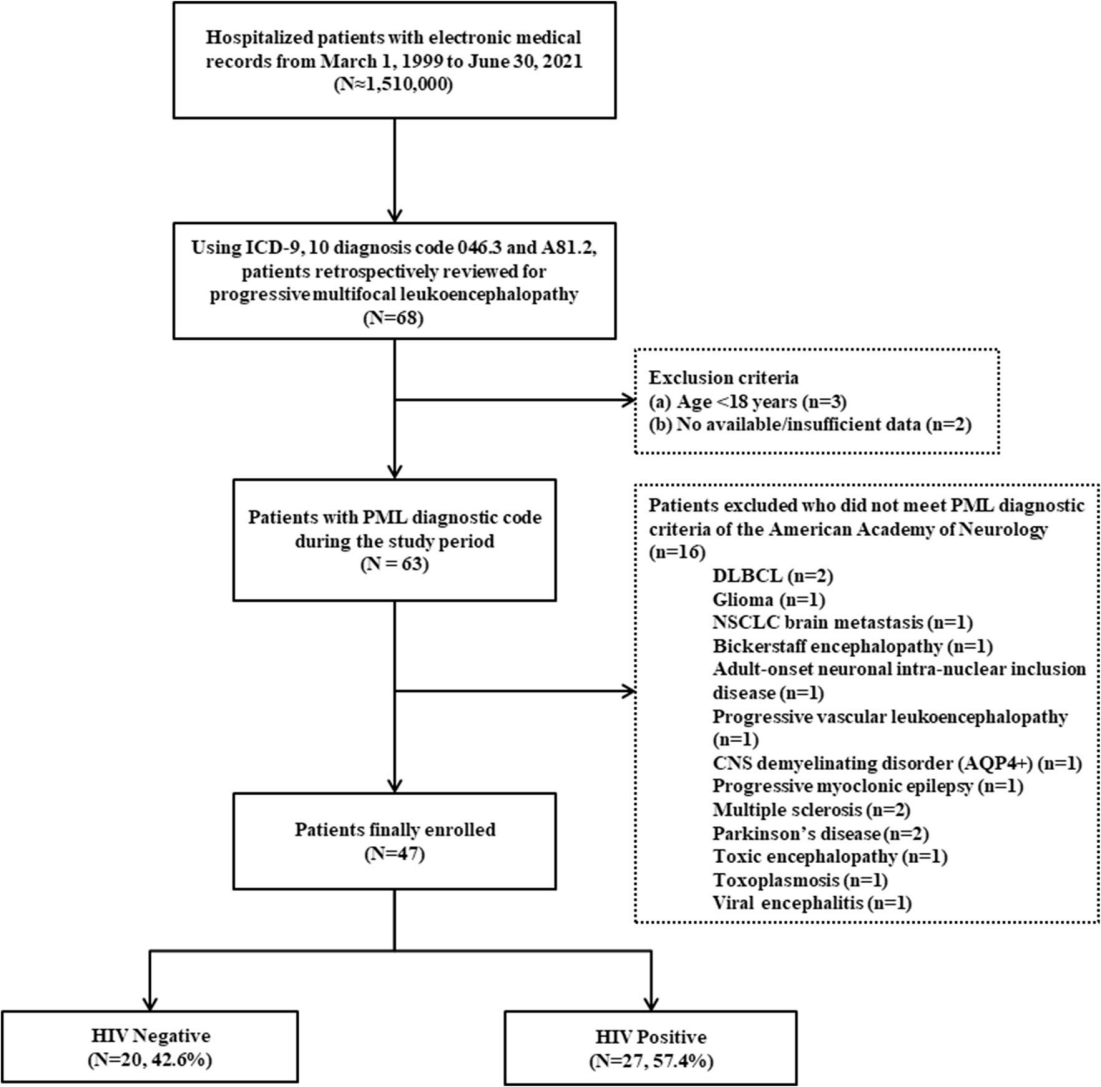
Flow chart depicting study population with progressive multifocal encephalopathy. *ICD* International Classification of Diseases, *PML* Progressive multifocal leukoencephalopathy, *HIV* Human immunodeficiency virus, *DLBCL* Diffuse large B cell lymphoma, *NSCLC* Non-small cell lung cancer, *CNS* Central nervous system, *AQP* Aquaporin.

Out of 68 patients with the PML diagnosis code, 47 patients meeting the American Academy of Neurology (AAN) diagnostic criteria were enrolled (Fig. 1)^19^. The median age was 46 years (interquartile range [IQR], 37–57), and 74.5% were male patients (Table 1). According to the AAN criteria, definite PML was observed in 44.7% of patients (21/47), while possible PML was observed in 55.3% (26/47). Of the 21 patients with a definite diagnosis of PML, eight had neuropathological demonstration of the typical histopathologic triad. The median initial mRS score was 4 (IQR, 3–4); 46.8% of patients had a mRS score of 4, and 8.5% had a mRS score of 5. Patients diagnosed with PML had a variety of immunosuppressive conditions; 27 (57.4%) were HIV positive, 7 (14.9%) had hematologic malignancies, 11 (23.4%) had solid organ cancer, 3 (6.4%) were diagnosed with rheumatologic disease, and 3 (6.4%) with solid organ transplant. Further review of concomitant medications revealed that 19 (40.4%) patients were using immunosuppressive drugs, and 10 (21.3%) had a history of chemotherapy.

**Table 1:** Baseline characteristics of patients with progressive multifocal leukoencephalopathy.

	Total (N = 47)	%
Age (years)	46 (37–57)	
Male sex (%)	35	74.5%
Diagnosis by AAN		
Definite	21	44.7%
Probable	0	0.0%
Possible	26	55.3%
Status (mRS)		
mRS_Initial	4 (3–4)	
mRS_2	7	14.9%
mRS_3	14	29.8%
mRS_4	22	46.8%
mRS_5	4	8.5%
Underlying disease (Risk factor)		
Human immunodeficiency virus	27	57.4%
Hematologic malignancy	7	14.9%
Lymphoma	4	8.5%
Leukemia	1	2.1%
Myelodysplasia	2	4.3%
Bone marrow/Stem cell transplant	3	6.4%
Solid organ cancer	11	23.4%
Rheumatologic disease	3	6.4%
Solid organ transplant	3	6.4%
Other diseases^a^	5	10.6%
Two or more underlying diseases	7	14.9%
Underlying medication		
Immunosuppressive agents^b^	19	40.4%
Chemotherapy^c^	10	21.3%
Other treatment^d^	7	14.9%

*AAN* American Academy of Neurology, *mRS* modified Rankin Scale.

^a^Other diseases include hypogammaglobulinemia, autoimmune cholangiopathy, psoriasis with monoclonal antibody treatment, and disseminated tuberculosis.

^b^Immunosuppressive agents include tacrolimus, corticosteroids, methotrexate, mycophenolate mofetil, and cyclosporine.

^c^Chemotherapeutic agents include adriamycin, bevacizumab, bortezomib, carboplatin, cisplatin, cyclophosphamide, cytarabine, etoposide, ifosfamide, irinotecan, melphalan, mitomycin, oxaliplatin, pazopanib, pomalidomide, rituximab, thiotepa, vincristine, 5-Fluorouracil, and tegafur/gimeracil/oteracil potassium.

^d^Other underlying medication treatments include ustekinumab, total body irradiation, and radiation therapy, in combination with an immunosuppressive or chemotherapeutic agent.

### Clinical features, neuroimaging, and laboratory data of patients with PML

The most frequently observed symptoms in patients with PML were motor weakness (51.1%), gait instability (46.8%), speech abnormalities (aphasia and dysarthria; 46.8%), and apraxia (42.6%) (Table 2). In brain magnetic resonance imaging, multifocal lesions were observed in 87.2% of patients, enhancement in 27.7%, posterior fossa involvement in 29.8%, and mass effect in 12.8%. The median neutrophil count was 3800 cells/µL (IQR, 1845–5935), median lymphocyte count was 785 cells/µL (IQR, 543–1618), median CD4 count was 66 cells/µL (IQR, 35–137), and the median neutrophil–lymphocyte ratio (NLR) was 3.07 (IQR, 2.48–15). The median HIV ribonucleic acid PCR titer in the HIV population was 118,000 copies/mL (IQR, 60450–392000). CSF opening pressure was 130 mm H_2_O (IQR, 93.8–156.3), and median CSF white blood cell count was 1 cell/µL (IQR, 0–2). The median time from symptom onset to PML diagnosis was 46 days (IQR, 28–91).

**Table 2:** Clinical manifestation, imaging and laboratory findings of patients with progressive multifocal leukoencephalopathy.

	Total (N = 47)	HIV		*p*-value
		Negative (n = 20, 42.6%)	Positive (n = 27, 57.4%)	
Presenting symptoms				
Aphasia	10 (21.3%)	6 (30.0%)	4 (14.8%)	0.286
Apraxia	20 (42.6%)	9 (45.0%)	11 (40.7%)	0.770
Diminished level of consciousness	12 (25.5%)	5 (25.0%)	7 (25.9%)	0.943
Dysarthria	12 (25.5%)	3 (15.0%)	9 (33.3%)	0.154
Gait instability	22 (46.8%)	11 (55.0%)	11 (40.7%)	0.333
Headache	8 (17.0%)	4 (20.0%)	4 (14.8%)	0.707
Hemiparesis	9 (19.1%)	4 (20.0%)	5 (18.5%)	0.999
Memory impairment	9 (19.1%)	5 (25.0%)	4 (14.8%)	0.465
Motor weakness	24 (51.1%)	13 (65.0%)	11 (40.7%)	0.100
Neglect	4 (8.5%)	2 (10.0%)	2 (7.4%)	0.999
Psychiatric symptom	7 (14.9%)	3 (15.0%)	4 (14.8%)	0.999
Seizure	9 (19.1%)	5 (25.0%)	4 (14.8%)	0.465
Sensory symptom	13 (27.7%)	2 (10.0%)	11 (40.7%)	**0.020**
Tremor	3 (6.4%)	0 (0.0%)	3 (11.1%)	0.251
Vertigo	5 (10.6%)	2 (10.0%)	3 (11.1%)	0.999
Vision change (Blind, Diplopia)	11 (23.4%)	2 (10.0%)	9 (33.3%)	0.086
MRI findings				
Multifocality of lesions	41 (87.2%)	18 (90.0%)	23 (85.2%)	0.999
Posterior fossa involvement	14 (29.8%)	4 (20.0%)	10 (37.0%)	0.207
Enhancement	13 (27.7%)	6 (30.0%)	7 (25.9%)	0.758
Mass effect	6 (12.8%)	3 (15.0%)	3 (11.1%)	0.999
Lab findings				
White blood cell (cells/µL)	5970 (4055–7313)	7070 (5970–8445)	4230 (3020–5525)	0.267
Hemoglobin (g/dL)	12.3 (10.2–13.8)	11.6 (10.1–13.1)	12.5 (11.5–13.5)	0.520
Platelet (cells/µL)	218 k (157 k-263 k)	219 k (200 k-321 k)	158 k (136 k-172 k)	0.707
Neutrophil (cells/µL)	3800 (1845–5935)	6480 (5265–7710)	2590 (1470–3675)	**0.008**
Neutrophil (%)	65.4% (53.7–78.7%)	91.0% (87.1–91.3%)	60.0% (53.8–68.7%)	** < 0.001**
Lymphocyte (cells/µL)	785 (543–1618)	340 (280–365)	830 (580–1530)	0.264
Lymphocyte (%)	18.9% (12.0–28.2%)	3.5% (3.3–5.7%)	26.3% (15.4–29.9%)	**0.004**
Neutrophil–lymphocyte ratio	3.07 (2.15–6.48)	6.70 (2.74–13.38)	2.20 (1.85–4.16)	**0.004**
CD4 (cells/µL)	66 (35.3–136.8)	43 (41.5–89.5)	45 (34.5–120.5)	0.606
CD8 (cells/µL)	430.5 (239.0–943.5)	163 (133.5–163.0)	352 (237–752.5)	0.378
HIV RNA PCR (copies/mL)			118,000 (60,450–392,000)	
CSF findings				
CSF_Opening pressure (mm H_2_O)	130 (93.8–156.3)	90 (75–115)	130 (115–152.5)	0.794
CSF_WBC (cells/µL)	1 (0–2)	0 (0–1)	0 (0–1.5)	0.339
CSF_RBC (cells/µL)	0 (0–4)	4 (2–427)	0 (0–1.5)	0.250
CSF_glucose (mg/dL)	62 (56–75)	117 (102.5–143.5)	61 (59–66)	**0.029**
CSF_protein (mg/dL)	45.1 (32.1–65.8)	31 (30.8–49.3)	37.4 (34.9–51.3)	0.342
John Cunningham virus				
CSF	15 (31.9%)	3 (15.0%)	12 (44.4%)	**0.032**
Blood	2/9 (22.2%)	1/5 (20.0%)	1/4 (25.0%)	0.999
From initial symptom onset to PML diagnosis (d)	46 (28–91)	42 (27–70)	55 (28–129)	0.090

Significant values are in bold.

*HIV* Human immunodeficiency virus, *RNA* Ribonucleic acid, *PCR* Polymerase chain reaction, *CSF* Cerebrospinal fluid, *PML* Progressive multifocal leukoencephalopathy, *MRI* Magnetic resonance imaging.

Sensory symptoms were more common in HIV patients than that in non-HIV patients (40.7% vs. 10.0%, *p* = 0.020); however, most other symptoms were not significantly different. In HIV patients, the neutrophil count was low (2590 [IQR, 1470–3675] vs. 6480 [IQR, 5265–7710], *p* = 0.008), whereas the lymphocyte ratio was high (26.3% [IQR, 15.4–29.9] vs. 3.5% [3.3–5.7%], *p* = 0.004) and consequently, lower NLR ratios were observed (2.20 [1.85–4.16] vs. 6.70 [2.74–13.38], *p* = 0.004). Furthermore, the detection rate of JCV (44.4% vs. 15.0%, *p* = 0.032) was higher in HIV patients (Table 2).

### PML-directed therapy

All HIV patients diagnosed with PML received HAART (Table 3). PML-directed therapy for the non-HIV patient group included immunosuppressant reduction in 25.0% of patients (5/20) and intravenous immunoglobulin administration in 20.0% (4/20). Of all patients with PML, 31.9% (15/47) were administered steroids, of whom 66.7% (10/15) were combined with other PML-directed therapy. Similarly, mirtazapine was used as PML-directed therapy in 27.7% (13/47) of all patients, and of these, 92.3% (12/13) were combined with other PML-directed therapy. Cidofovir and mefloquine were administered to 10.6% (5/47) and 8.5% (4/47) patients, respectively.

**Table 3:** Treatment and survival outcomes of patients with progressive multifocal leukoencephalopathy.

	Total (N = 47)	HIV		*p*-value
		Negative (n = 20, 42.6%)	Positive (n = 27, 57.4%)	
PML directed therapy				
HAART			27 (100%)	
Reduced immunosuppression	5 (10.6%)	5 (25.0%)	0 (0.0%)	0.010
Cidofovir	5 (10.6%)	2 (10.0%)	3 (11.1%)	0.999
Mirtazapine	13 (27.7%)	7 (35.0%)	6 (22.2%)	0.333
Mefloquine	4 (8.5%)	2 (10.0%)	2 (7.4%)	0.999
Checkpoint_inhibitor_therapy	1 (2.1%)	1 (5.0%)	0 (0.0%)	0.426
Intravenous immunoglobulin	4 (8.5%)	4 (20.0%)	0 (0.0%)	0.027
Steroid	15 (31.9%)	9 (45.0%)	6 (22.2%)	0.098
Others	1 (2.1%)	1 (5.0%)	0 (0.0%)	0.426
Overall mortality	27 (57.4%)	16 (80.0%)	11 (40.7%)	**0.007**
1-year mortality	19 (40.4%)	12 (60.0%)	7 (25.9%)	**0.019**
90-day mortality	9 (19.1%)	5 (25.0%)	4 (14.8%)	0.465
30-day mortality	5 (10.6%)	3 (15.0%)	2 (7.4%)	0.638
IRIS	9 (19.1%)	0 (0.0%)	9 (33.3%)	0.006
mRS_last follow-up	4 (3–6)	5 (4–6)	4 (2–5)	**0.020**
mRS_2	9 (19.1%)	1 (5.0%)	8 (29.6%)	
mRS_3	5 (10.6%)	2 (10.0%)	3 (11.1%)	
mRS_4	13 (27.7%)	5 (25.0%)	8 (29.6%)	
mRS_5	8 (17.0%)	5 (25.0%)	3 (11.1%)	
mRS_6	12 (25.5%)	7 (35.0%)	5 (18.5%)	
mRS_improved	7 (14.9%)	0 (0.0%)	7 (25.9%)	**0.015**
Duration of survival (d)	608 (94–2391)	184 (74–1566)	1564 (254–3444)	0.052

Significant values are in bold.

*HIV* Human immunodeficiency virus, *HAART* Highly active antiretroviral therapy, *IRIS* Immune reconstitution inflammatory syndrome, *mRS* modified Rankin Scale.

### Survival outcomes

**Figure 2:**
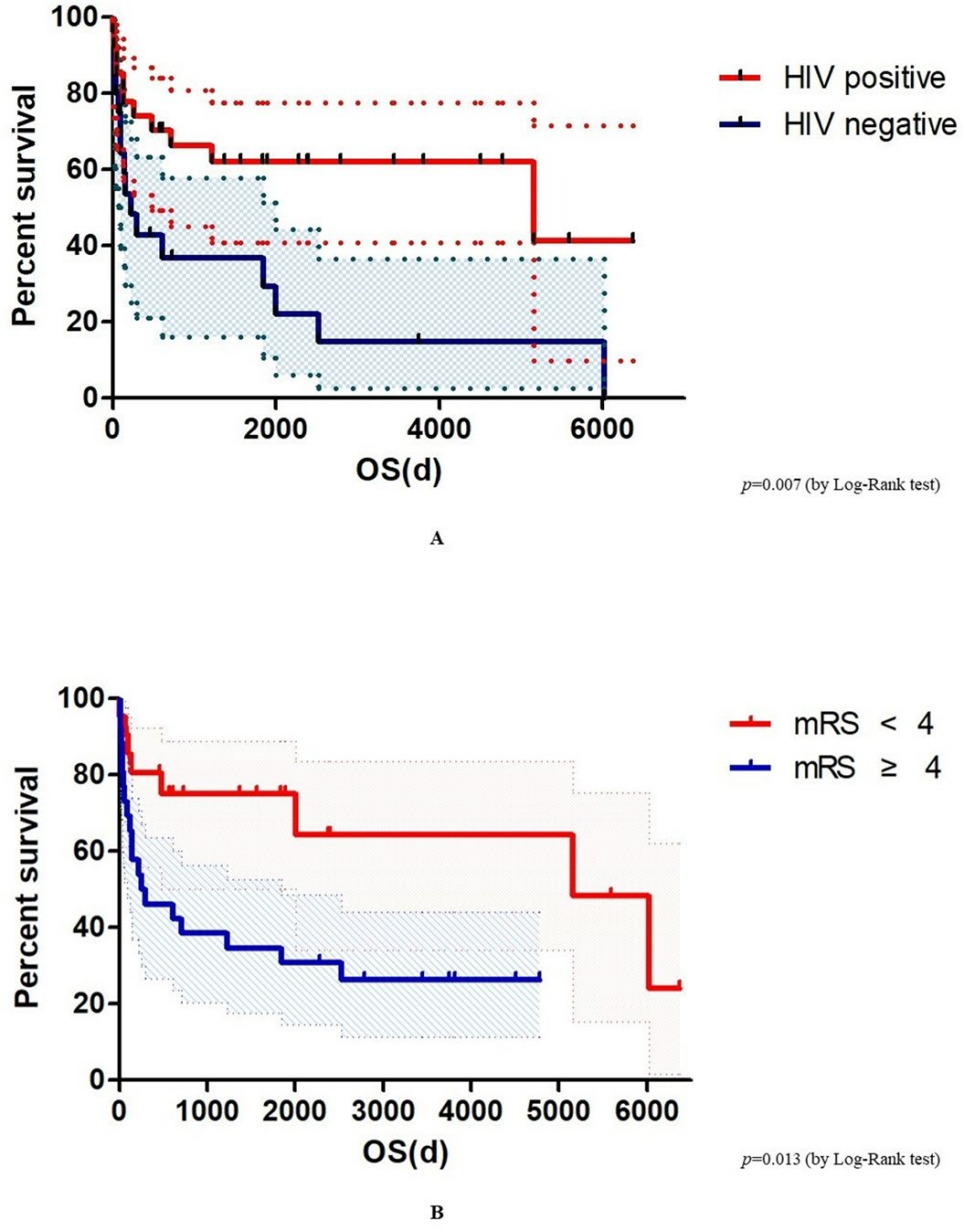
Kaplan-Meier curve for overall mortality in patients with progressive multifocal leukoencephalopathy according to (**A**) HIV infection and (**B**) mRS score. *HIV* human immunodeficiency virus, *mRS* modified Rankin Scale, *OS* overall survival.

The median follow-up duration was 608 days (IQR, 94–2391) (Table 3). Median survival was longer in the HIV patient group than in the non-HIV patient group (1564 days [IQR, 254–3444] vs. 184 days [IQR, 74–1566], *p* = 0.052). The 30-day, 90-day, and 1-year mortality rates were overall higher in the non-HIV group, with the 1-year mortality rate being statistically significant (60.0% vs. 25.9%, *p* = 0.019). The overall mortality rate was also significantly higher in the non-HIV group (80.0% vs. 40.7%, *p* = 0.007), which was further confirmed by the Kaplan–Meier curve and log-rank test (*p* = 0.007) (Fig. 2A).

The median mRS score at the last follow-up was 4 (IQR, 3–6) (Table 3). Of 47 PML patients, 19.1% had mRS score of 2, 10.6% had mRS score of 3, 27.7% had mRS score of 4, 17.0% had mRS score of 5, and 25.5% had mRS score of 6 at the last follow-up. The median last follow-up mRS score was higher in the non-HIV patient group than that in the HIV group (5 [IQR, 4–6] vs. 4 [IQR, 2–5], *p* = 0.020). Patients with moderately severe disability with 4 points or higher mRS, unable to walk without assistance, were 85.0% in the non-HIV group and 59.2% in the HIV group. All patients with improved mRS score during follow-up period were HIV PML patients (0% vs. 25.9%, *p* = 0.015). Significant differences in overall mortality according to the mRS cutoff point 4 were confirmed by the Kaplan–Meier curve and log-rank test (*p* = 0.013) (Fig. 2B).

### Univariable and multivariable analyses of overall mortality

In the univariable analysis, initial mRS score (HR 1.719, 95% CI 1.030–2.868, *p* = 0.038) was associated with increased overall mortality (Table 4). Likewise, in the multivariable model, initial mRS score (HR 1.685, 95% CI 1.028–2.762, *p* = 0.038) was related to increased overall mortality, and HAART (HR 0.374, 95% CI 0.172–0.815, *p* = 0.013) was associated with decreasing overall mortality (Table 4).

**Table 4:** Univariable and multivariable analyses of overall mortality in patient with progressive multifocal leukoencephalopathy by Cox Proportional-Hazards Model.

Characteristics	N	Univariable analysis			Multivariable analysis		
		HR	95% CI	*p*-value	HR	95% CI	*p*-value
Age		1.019	0.989–1.049	0.228			
Sex							
Male	35	1					
Female	12	1.495	0.608–3.672	0.381			
Initial mRS		1.719	1.030–2.868	**0.038**	1.685	1.028–2.762	**0.038**
HIV with HAART	27	0.677	0.240–1.912	0.461	0.374	0.172–0.815	**0.013**
IRIS	9	0.462	0.098–2.173	0.328			

Significant values are in bold.

*mRS* modified Rankin Scale, *HIV* Human immunodeficiency virus, *HAART* Highly active antiretroviral therapy, *IRIS* Immune reconstitution inflammatory syndrome.

## Discussion

With the recent increase in organ transplantation and immunosuppressive drug usage, PML has been reported in various disease groups, including not only HIV infection but also lymphoproliferative disorders, post-organ transplantation, and autoimmune diseases^1,20^. In our study, we described the long-term prognosis and the factors influencing long-term overall mortality of patients with PML under the introduction of HAART and various immunosuppressive conditions.

In previous studies, the median survival duration increased up to 66 months after the introduction of HAART in the HIV population; however, the overall mortality rate was 74.5–78.0%^1,9^. In the non-HIV population, the median survival duration of two months was reported, and the case-fatality rate was 90%^10^. In our study, median survival duration was 1,564 days (IQR 254–3444) in the HIV group and 184 days (IQR 74–1566) in the non-HIV group. Additionally, the overall mortality rate of patients with PML during the long observational period was 40.7% in the HIV group and 80.0% in the non-HIV group. Although advances in treatment options have been considered for only a few patients, the prognosis of PML has improved compared to the previous decade^4,8^. As the introduction of HAART had a significant effect on the median survival of PML patients in HIV population, it is likely that changes in HAART initiation regardless of CD4 count will further improve the prognosis of PML.

Various studies have attempted to elucidate predictors of prognosis and mortality in patients with PML. Khanna et al. showed that baseline CD4 + T cell count (HR 0.52, *p* = 0.010) and HAART (HR 0.37, *p* = 0.006) were associated with overall mortality of patients with PML^2^. In a similar report, a higher baseline CD4 + T cell count (HR 0.33) and CSF inflammatory profile (HR 0.12) were significantly related to the long-term survival of patients with PML^21^. Another report by Koralnik et al. showed an association between JCV-specific cytotoxic T-lymphocytes (CTLs) and HIV-positive PML survivors and HIV-negative PML patients with improving clinical status^22^. In a comparative study, Marzocchetti reported that JCV-specific CTLs were associated with a trend toward more prolonged survival in patients with PML^23^. Our study showed that higher initial mRS score was a significant risk factor for overall mortality in a long-term follow up period. In addition, the introduction of HAART was a predictor of long-term survival in HIV-positive PML patients.

The first stage of JCV infection is an asymptomatic, persistent infection of the kidneys by the nonpathogenic JCV that occurs in the majority of the general population^24^. During this stage, the virus may establish a latent infection in other secondary sites, including lymphoid tissues, bone marrow, and possibly the brain^24^. These asymptomatic latent infections can lead to PML by impairment of cellular immunity due to a particular underlying disease and immunosuppressive drug usage^24^. As a result, PML treatment proceeds in two directions: immune reconstitution or antiviral therapy. Drugs such as mefloquine and cidofovir, which have a direct mechanism of action against JCV infection and replication, have not shown clinically meaningful results; therefore, PML treatment mainly involves reconstitution of the immune system^16,17,25^. Immune reconstitution can be achieved through HAART initiation in patients with HIV; however, it is difficult to achieve in other immunosuppressive conditions. Hematologic malignancy is often pathophysiologically related to immune disruption and is accompanied by transplantation or high-dose chemotherapy^10^. In addition, immunosuppressive reduction therapy is a significant burden when immunosuppressive agents are required to modulate disease activity, such as in solid organ transplantation or rheumatic disease.

HAART is the mainstream treatment for HIV-positive patients with PML. Various studies have reported a decrease in the incidence and mortality of PML in HIV-positive patients after the introduction of HAART. In the Eurosida cohort, the incidence of PML decreased from 0.7 to 0.07 per 100 cases per year (PY) after the introduction of HAART^26^. Similarly, Engsig et al. showed that the median survival of HIV-positive patients with PML increased from 0.4 to 1.8 years after the introduction of HAART^9^. In another study, the 1-year mortality due to PML in patients with HIV decreased from 82.3 per 100 cases PY in the pre-HAART era to 37.6 per 100 cases PY in the HAART era^2^. In a multivariable model of 186 patients with PML, the overall mortality was associated with HAART in patients with HIV (HR 0.37, *p* = 0.006)^2^. In our study, patients with HIV with HAART had a 63% reduction in overall mortality in PML compared to non-HIV patients. Therefore, HAART (HR 0.374, *p* = 0.013) in HIV patients was associated with decreasing overall mortality or long-term survival of PML patients.

PML may rarely occur in HIV patients receiving continuous HAART^27^. However, in this study, 29.6% (8 of 27) of HIV patients were diagnosed with PML during HAART. The 1-year mortality rate in the group receiving HAART was lower than that in the group newly starting HAART (12.5% vs. 31.6%, *p* = 0.311); however, no statistical difference was observed (Supplementary Table 1). Additionally, the median survival duration of the group receiving HAART was longer (1921 days [799–4995] vs. 1,221 days [126–2786], *p* = 0.243). This suggests that the prognosis may be better in HIV patients receiving HAART (who recovered some immune response) than in naive HIV patients. However, the factors contributing to the disease course of PML in HIV patients receiving HAART are unknown. Genetic risk factors, such as mutations in VP1 or polymorphisms in the tumor suppressor protein p53, may affect the pathogenesis of PML^28,29^. In this regard, a prospective study is required to analyze and compare the immunological characteristics of the two groups.

Our study also showed that the initial mRS score is an important prognosis factor, and in particular, the long-term prognosis is poor in the case of mRS score of 4 or higher through the Cox regression model. PML was dominated by motor weakness (51.1%), gait instability (46.8%), speech abnormalities (46.8%), and apraxia (42.6%), but various symptoms may appear depending on the site of involvement. Since PML can occur not only in the case of hematologic malignancy (14.9%) and HIV infection (57.4%) but also in various disease groups taking immune-modulating agents, its diagnosis is difficult and time-consuming. Early detection contributes to a better prognosis of PML with limited disease progression and rapid and effective immune reconstitution^24^. PML pathophysiology involves oligodendrocyte destruction and subsequent demyelination of the CNS^1^. Currently, treatments that can reverse CNS destruction and subsequent demyelination are unavailable, so early recognition of possible PML and achieving immune reconstitution are crucial for improving prognosis.

In this study, the median lymphocyte count was 785 cells/microliter, which was not as severe as expected. This may be due to the fact that HIV-negative patients had a much lower baseline lymphocyte rate of 3.5% (3.3–5.7%), while HIV-positive patients had a baseline lymphocyte rate of 26.3% (15.4–29.9%) and 830 (580–1530). PML was also observed in patients with already recovered lymphocytes, with eight patients taking HAART and nine patients suffering from immune reconstitution inflammatory syndrome (IRIS). Lymphopenia is a risk factor for PML, but monitoring of absolute lymphocyte counts alone is not an accurate predictor of risk because it does not take into account the complexity and diversity of the immune system^30^. The fact that dimethyl fumarate-associated PML typically occurs in the context of severe lymphopenia, whereas abnormal production of IL-10, expression of PD-1, and reduced expression of CD49d by JCV-specific T cells are observed in natalizumab-induced PML, suggests that not only the number of lymphocytes but also their composition is important in the pathogenesis of PML^30–32^. In addition, chronic infection with persistent antigens can lead to immune exhaustion, eventually rendering T cells unable to respond effectively to persistent antigens^33^. CD8 + T cells are specialized for intracellular pathogen clearance, but a combination of decreased effector function, inhibitory receptor expression, and cytokine hyporesponsiveness can lead to CD8 + T cell exhaustion in chronic viral infections^34,35^. CD4 + T cells are essential for the immune response to chronic viral infection, but due to decreased production of IL-2, TNFα, and IFNγ and increased expression of the suppressive cytokine IL-10, CD4 + T cells exhibit altered function during chronic infection, which is considered a form of functional exhaustion^35–37^.

Our study had several strengths. The long-term prognosis was evaluated while fully reflecting the introduction of HAART and the recommendation of antiretroviral treatment regardless of CD4 cell count in randomized controlled trials such as Temprano ANRS 12,136 and START^38,39^. In addition, the long-term prognosis was evaluated by reflecting the overall PML occurring under various immunosuppressive conditions through increased use of immunosuppressive agents. For patients with hopeless discharge or loss of follow-up, accurate mortality data could be obtained from the Ministry of the Interior and Safety of South Korea. This allowed us to accurately assess mortality or long-term prognosis, the primary or secondary endpoint of our study.

Our study has certain limitations. Due to the retrospective nature of this study, PML diagnosis or treatment may have physician-dependent selection bias. Furthermore, there were some cases where brain biopsy could not be performed depending on the patient's condition; the diagnosis was made based on clinical and radiographic findings. In addition, due to the low prevalence of PML, the sample size was small despite the long observation period. Therefore, to overcome this low prevalence and incidence, our team is planning a nationwide PML study through a multicenter study or anonymized data linkage.

## Conclusion

With the widespread adoption of HAART, the survival duration of HIV-positive patients with PML has extended. However, the mortality rate and the prognosis for PML in non-HIV patients remain frustrated. Initial mRS score is a significant risk factor for long-term overall mortality in patients with PML. Early detection of PML and early initiation of HAART in patients with HIV may improve prognosis.

## Materials and methods

### Patient selection

We screened approximately 1.51 million hospitalized patients with electronic medical records who were admitted to two tertiary hospitals with 2,400 and 1,000 beds from March 1999 to June 2021. Of these, we retrospectively reviewed patients with PML using International Classification of Diseases-9, 10 diagnostic codes 046.3 and A81.2. Eligibility criteria included age > 18 years and patients with PML diagnosed by the AAN criteria. Excluded patients were those with insufficient clinical data (n = 2) and who did not meet PML diagnostic criteria, such as diffuse large B-cell lymphoma (n = 2), glioma (n = 1), non-small cell lung cancer brain metastasis (n = 1), Bickerstaff encephalopathy (n = 1), adult-onset neuronal intra-nuclear inclusion disease (n = 1), progressive vascular leukoencephalopathy (n = 1), CNS demyelinating disorder (aquaporin-4) (n = 1), progressive myoclonic epilepsy (n = 1), multiple sclerosis (n = 2), Parkinson’s disease (n = 2), toxic encephalopathy (n = 1), toxoplasmosis (n = 1), and viral encephalitis (n = 1) (Fig. 1). A total of 47 patients with PML were finally enrolled.

This study was approved by the Institutional Review Boards (IRBs) of Yonsei University College of Medicine (IRB no. 4-2021-1045). Informed consent was waived by the IRBs of Yonsei University College of Medicine due to the retrospective nature of the study. This study complied with the Declaration of Helsinki and Good Clinical Practice guidelines.

### Definition of variables

PML was defined according to the AAN diagnostic criteria with clinical features, neuroimaging, pathology, and laboratory data^19^. Its diagnostic classification includes definite, probable, and possible PML^19^. Definite PML diagnosis is confirmed by neuropathologic demonstration of the typical histopathologic triad (demyelination, bizarre astrocytes, and enlarged oligodendroglial nuclei) coupled with the presence of JCV, or typical clinical and radiographic features with JCV detection^19^. Probable PML is based on either histopathologic triad with no JCV detection or JCV polymerase chain reaction (PCR) positivity in the cerebral spinal fluid (CSF) with the absence of clinical features or neuroimaging^19^. Possible PML diagnosis requires the presence of JCV without the typical histopathologic triad, or clinical and radiological features with negative CSF JCV PCR, or positive CSF JCV PCR without clinical and radiological features^19^. The degree of neurological disability was measured with the modified Rankin Scale (mRS) at each clinical follow-up^40^. IRIS is an inflammatory process that occurs through immune reconstitution during HAART in HIV patients that cannot be explained by new opportunistic infection or drug toxicity^41^.

Immunosuppressive agents include tacrolimus, corticosteroids, methotrexate, mycophenolate mofetil, and cyclosporine. Chemotherapeutic agents include adriamycin, bevacizumab, bortezomib, carboplatin, cisplatin, cyclophosphamide, cytarabine, etoposide, ifosfamide, irinotecan, melphalan, mitomycin, oxaliplatin, pazopanib, pomalidomide, rituximab, thiotepa, vincristine, 5-Fluorouracil, and tegafur/gimeracil/oteracil potassium. Other underlying medication treatments include ustekinumab, total body irradiation, and radiation therapy, used in combination with immunosuppressive or chemotherapeutic agents.

The mortality data were obtained from the Ministry of the Interior and Safety of South Korea, which collects mortality data of all Korean citizens.

### Clinical outcomes

The primary endpoint of this study was overall mortality during the observational period. Overall mortality was defined as death from any cause. The secondary endpoints were 30-day mortality, 90-day mortality, 1-year mortality, and mRS score of the last follow-up visit.

### Statistical analysis

Comparison between groups was performed using the Chi-squared and Fisher’s exact tests for categorical variables and the Mann–Whitney U test for continuous variables. The *p*-value of < 0.05 was considered statistically significant. A Kaplan–Meier curve was drawn using patient survival data from the time of admission to either death or the last follow-up date. The log-rank test was used to determine the difference in survival distributions between the two groups. Hazard ratio (HR) and 95% confidence intervals (CIs) of variables for overall mortality were analyzed using a Cox proportional hazards model. Variables for multivariable analysis were selected based on the clinically significant risk factors in univariable analysis. Statistical analysis was performed using IBM SPSS Statistics for Windows version 26 (IBM Corp., Armonk, NY, USA).

### Ethics declarations

This study was approved by the Institutional Review Boards (IRBs) of Yonsei University College of Medicine (IRB no. 4-2021-1045). Informed consent was waived due to the retrospective nature of the study. This study complied with the Declaration of Helsinki and Good Clinical Practice guidelines.

### Supplementary Information

Supplementary Table 1.

## Data Availability

The datasets generated during and/or analyzed during the current study are available from the corresponding author on reasonable request.
