# Supplementary material for: Long-term prognosis and overall mortality in patients with progressive multifocal leukoencephalopathy
Source: Sci Rep. 2023 Aug 31;13:14291. doi: 10.1038/s41598-023-41147-9 (PMC10471597; doi:10.1038/s41598-023-41147-9)
Supplement: Supplementary file 1 — Supplementary Table 1. [file 41598_2023_41147_MOESM1_ESM.docx]

| **Supplementary Table 1. Subgroup analysis of HIV patients with progressive multifocal leukoencephalopathy** | | | | |
| --- | --- | --- | --- | --- |
|  |  |  |  |  |
|  |  | **HIV** | | ***p-*value** |
|  | **Total (N=27)** | **Dx (off HAART) (n=19, 70.4%)** | **Dx (on HAART) (n=8, 29.6%)** |  |
| Age (years) | 40 (34-47) | 39.0 (33.0-46.5) | 40.5 (38.5-46.0) | 0.807 |
| Male sex (%) | 23 (85.2%) | 16 (84.2%) | 7 (87.5%) | 0.999 |
| Diagnosis by AAN |  |  |  |  |
| Definite | 15 (55.6%) | 12 (63.2%) | 3 (37.5%) | 0.398 |
| Possible | 12 (44.4%) | 7 (36.8%) | 5 (62.5%) |  |
| mRS_Initial | 3 (3-4) | 4 (3-4) | 3 (3-4) | 0.799 |
| CD4 | 70 (34-149) | 47 (33-104) | 202 (67-522) | 0.103 |
| HIV RNA PCR (copies/mL) | 63900 (436-375500) | 70867 (41149-387000) | 19733 (33-486550) | 0.779 |
| Log (HIV RNA) | 4 (2-5) | 4 (4-5) | 3 (1-4.5) | 0.121 |
| JCV_CSF | 12/20 (60.0%) | 11/16 (68.8%) | 1/4 (25.0%) | **0.043** |
| MRI findings |  |  |  |  |
| Multifocality of lesions | 23 (85.2%) | 17 (89.5%) | 6 (75.0%) | 0.558 |
| Posterior fossa involvement | 10 (37.0%) | 8 (42.1%) | 2 (25.0%) | 0.666 |
| Enhancement | 7 (25.9%) | 1 (5.3%) | 6 (75.0%) | **0.001** |
| Mass effect | 3 (11.1%) | 3 (15.8%) | 0 (0.0%) | 0.532 |
| mRS_last follow-up | 4 (2-5) | 4 (4-6) | 2 (2-3) | **<0.001** |
| 30-day mortality | 2 (7.4%) | 2 (10.5%) | 0 (0.0%) | 0.349 |
| 90-day mortality | 4 (14.8%) | 4 (21.1%) | 0 (0.0%) | 0.168 |
| 1-year mortality | 7 (25.9%) | 6 (31.6%) | 1 (12.5%) | 0.311 |
| Overall mortality | 11 (40.7%) | 9 (47.4%) | 2 (25.0%) | 0.289 |
| IRIS | 9 (33.3%) | 4 (21.1%) | 5 (62.5%) | 0.072 |
| Duration of survival (d) | 1564 (254-3444) | 1221 (126-2786) | 1921 (799-4995) | 0.243 |

Abbreviations: HIV, Human immunodeficiency virus; HAART, Highly active antiretroviral therapy; AAN, American Academy of Neurology; mRS, modified Rankin Scale; RNA, Ribonucleic acid; PCR, Polymerase chain reaction; JCV, John Cunningham virus; CSF, Cerebrospinal fluid; MRI, Magnetic resonance imaging; IRIS, Immune reconstitution inflammatory syndrome
